# A Culturally Tailored Psychoeducation Group for Reducing Mental Health Stigma of African American Women and their Social Support

**DOI:** 10.1007/s10597-025-01537-x

**Published:** 2025-10-24

**Authors:** Courtney Williams, LaKaylyn Washington, Brian McCabe, Katilya Ware

**Affiliations:** Auburn University, Auburn, United States

**Keywords:** Mental health stigma, Perinatal mood and anxiety disorders, Severe maternal morbidity and mortality, African American women, Community engagement

## Abstract

This engagement award project evaluated the preliminary effectiveness of a culturally tailored psychoeducation session aimed at reducing mental health stigma among African American women and their social support networks. Participants included 25 community members, including African American women of reproductive age and members of their social support networks, engaged in a two-hour psychoeducation group session. This session addressed perinatal mood disorders, cultural and historical factors contributing to mental health stigma, impacts of untreated conditions, and culturally appropriate resources. Participants completed the Stigmatized Attitudes Toward Mental Illness Scale (SATMIS) before and after the psychoeducation session, along with a satisfaction questionnaire. Analysis revealed a large (Cohen’s *d* = 0.98), significant reduction in stigmatized attitudes toward mental illness from pre-session (*M* = 62.4, *SD* = 10.2) to post-session (*M* = 48.9, *SD* = 9.7; *p* < .001). Most (92%) participants reported high satisfaction with the psychoeducation session. This engagement project provides preliminary evidence that a brief, culturally tailored psychoeducation group session can effectively reduce mental health stigma among African American women and their support networks. Given the disproportionate burden of perinatal mood and anxiety disorder (PMADs) and maternal mortality among African American women, this approach shows potential for addressing a significant barrier to mental health care utilization in this population.

## Background

African American women are three to four times more likely to die from pregnancy-related causes compared to white women, indicating a significant gap in maternal mortality rates (Hoyert, 2023). Perinatal mood and anxiety disorders (PMADs) represent the most common complication of pregnancy and one of the leading causes of maternal mortality, accounting for 40% of deaths between 2016 and 2017 (Craemer et al., 2023). African American women are disproportionately affected by these conditions, being twice as likely to experience perinatal mood and anxiety disorders compared to white women (Howell et al., 2005; Pao et al., 2019). Despite the higher prevalence, African American women are 50% less likely to receive mental health treatment compared to White women (Kozhimannil et al., 2011).

African American women have identified multiple sources of stigma that inhibit their help-seeking behaviors, including a lack of culturally responsive care, mistrust in mental health care and its effectiveness based on cultural relatedness, and the assumption that needing assistance jeopardizes one’s strength (Nelson et al., 2021). The Strong Black Woman schema (SBW) is one culturally sensitive theory relevant for African American maternal mental health, help-seeking behaviors, stigma, and treatment. This schema points to what is known as the “Superstrong Black Mother” highlighting a controlling image impacting motherhood experiences for some African American women. This image states that good mothering for African American women means being strong and solely responsible for their children. Living up to this idealistic image can be an emotionally taxing reality for African American mothers coping with structural inequities throughout their life, hindering from seeking help for concerns such as perinatal anxiety and/or depression (Brantley, 2023; Elliott & Reid, 2016; Nelson et al., 2023).

Psychoeducation rooted in cultural sensitivity has been associated with reduced stigmatizing attitudes and increased seeking of mental health treatment among African American women (Alvidrez et al., 2010). Cultural tailoring of interventions is crucial, as research suggests that approaches that incorporate cultural values and address specific barriers faced by marginalized populations are more effective (Conneely et al., 2023). The inclusion of social or community support (family, friends, religious leaders) in programming and interventions has also been associated with increased use of mental health services among African American women (Pickard et al., 2011).

While there has been an increase in attention towards racial disparities in maternal mortality of African American women, few interventions specifically address the role of mental health stigma during the perinatal period for African American women and their social support networks. The current engagement project identified the effects of a culturally tailored psychoeducation group for African American women and their community supports.

## Methods

### Participants

A total of 25 community members attended this psychoeducation session, including African American women of reproductive age and members of their social support networks (i.e., parents, partners, faith-based leaders, and health experts). Participation in this session occurred as part of a larger grant project training African American women and their support networks to engage in patient-centered maternal health research around various topics, including PMADs. Community members were invited to participate through partnerships with community-based organizations, healthcare settings, and predominantly African American churches. This engagement project was reviewed and approved by the XX University Institutional Review Board (protocol #23 − 007). All participants provided informed consent prior to participation and were informed about the purpose of the engagement project, data collection procedures, confidentiality protections, and the voluntary nature of their participation.

### Psychoeducation Group

This psychoeducational group intervention was developed using a community engagement approach guided by the “Equity and Inclusion Guiding Engagement Principles” developed by the Advisory Panel on Patient Engagement (PEAP) (PEAP,^2021^). These principles emphasize inclusion of diverse perspectives, equitable partnerships, trust and trustworthiness, and accountability in research engagement for developing strong patient-centered research teams and studies. The broader engagement project applied these principles by forming a leadership team comprising community stakeholders in maternal health (i.e., African American Women who recently gave birth, nurses, mental health professionals, faith-based leaders) who collaboratively created the session structure and content based on stakeholder-driven aims and best available literature on perinatal mental health, help seeking, and stigma reduction of African American women. The two-hour psychoeducation group session was designed to (1) increase awareness of perinatal mood and anxiety disorders, (2) address cultural and historical factors that contribute to mental health stigma among African American women, (3) highlight the impact of untreated mental health conditions on maternal and infant health, and (4) provide information about culturally-appropriate mental health resources and treatment. The session also incorporated important cultural elements identified by African American women in current literature, such as recognition of historical trauma and medical racism affecting African American women’s healthcare experiences, acknowledgment of cultural strengths, including spirituality, resilience, and community support, and discussion of the “strong Black woman” stereotype and its impact on help-seeking (Johnson et al., 2020; Nelson et al., 2021; Nelson et al., 2023; Prather et al., 2018). The session was facilitated by two African American women, both doctoral students with at least three years of training in counseling psychology. These facilitators brought relevant expertise from their previous work on the Strong Black Women Schema, racial health disparities, and maternal health.

### Measures

Mental health stigma was assessed using the Stigmatized Attitudes Toward Mental Illness Scale (SATMIS; Kobau et al., 2010): A 28-item instrument capturing three dimensions: social distance, stereotype endorsement, and perceptions of mental illness. Higher scores reflect greater stigmatization of attitudes toward mental illness. Community members completed the SATMIS before the start of the session and again immediately after the conclusion of the two-hour psychoeducation session.

For additional feedback on the psychoeducation session, the engagement team developed an 8-item questionnaire to assess participant’s experiences. Participants rated their satisfaction on a 5-point Likert-type scale ranging from 1 (strongly disagree) to 5 (strongly agree), with higher scores indicating greater appreciation of the session. The questionnaire captured perspectives on content relevance, delivery approach, and perceived value of the information presented.

### Analysis Plan

Changes in SATMIS scores from pre- to post- session were analyzed using a Generalized Linear Model in SPSS with a Cohen’s *d* effect size. A Generalized Linear Model chosen to accommodate the possibility of non-normal distribution of scores and to provide more robust parameter estimates. A pre-posttest design was selected to allow for efficient measurement of changes while minimizing participant burden in a community-based setting. Descriptive statistics, including mean and frequencies, were used to analyze participants’ satisfaction with the psychoeducation session and content.

## Results

Analysis of pre- and post-session SATMIS scores revealed a large-sized, statistically significant reduction in stigmatized attitudes toward mental illness. The mean pre-intervention SATMIS score was 62.4 (*SD* = 10.2), while the mean post-intervention score was 48.9 (*SD* = 9.7), indicating a reduction over time in stigmatized attitudes toward mental illness among African American women and their community support (*b* = 0.32, *p* <.001, *95%* CI [0.16, 0.48], Cohen’s *d* = 0.98).

All content was successfully delivered in under two hours. High levels of participant satisfaction were reported (*M* = 4.83), with 92% of participants indicating that the information presented was valuable and insightful.

## Discussion

The findings from this engagement project suggest that a culturally tailored psychoeducation group session is both feasible and effective in reducing stigmatized attitudes toward mental illness among African American women and their social support networks. The large effect size observed (Cohen’s d = 0.98) is particularly promising, especially when compared to similar mental health stigma reduction interventions; a systematic review (Thornicroft et al., 2016) of anti-stigma interventions found most educational approaches achieved only small to moderate effects (Cohen’s *d* = 0.28–0.58). Our results align with findings that culturally adapted, or culturally sensitive interventions are more effective for racial minority populations than generic approaches (Ponting et al., 2020).

The preliminary effectiveness of this psychoeducation group session suggests a useful approach to mitigating poor maternal health outcomes among African American women. This aligns with research demonstrating how culturally responsive mental health interventions can significantly improve maternal health trajectories among minoritized women (Lara-Cinisomo et al., 2018). By including support persons in the session, we address not only individual-level stigma but also stigma within the immediate social network, which has been identified as a significant barrier to help-seeking (Feinberg et al., 2022). That is, the session’s effectiveness for both African American women of reproductive age and their support persons suggests that this approach can help build supportive environments for African American women experiencing perinatal mental health challenges.

The responses revealed high levels of participant satisfaction, indicating that community members found the session and information valuable. Our integration of culturally specific factors, particularly the Strong Black Woman Schema, appeared especially meaningful to participants. These results align with literature identifying cultural relevance and community engagement as critical factors in intervention acceptability among African American women (Goodman et al., 2020). Our work aligns with recommendations by Watson-Singleton and colleagues (2019), who indicated that culturally specific elements and acknowledgment of the unique stressors faced by African American women may contribute to participant acceptability and satisfaction within a community-based mental health program.

## Limitations

This engagement project had several limitations to consider. The relatively small sample size limits the generalizability of our findings, particularly as participant demographic information was not collected due to this psychoeducation session and data collection being part of an engagement project focused primarily on program implementation rather than pilot research. Future engagement projects and studies should examine how various factors such as age, education level, or specific relationship to the pregnant woman might influence the effectiveness of this psychoeducation group session. It should be noted that study participants were also enrolled in a mental health engagement project, so they may have been more open to participating in a group intervention to improve mental health and reduce stigmatizing beliefs. Future research should recruit participants from the general population to ensure generalizability of findings.

This project also relied on a single-arm, pre-post assessment. While this approach showed changes in stigmatized attitudes, future studies should add a longer follow-up assessment period to provide evidence about whether changes in stigmatized perceptions are maintained over time. Future work should also assess whether these attitudinal shifts translate to actual help-seeking behaviors or treatment utilization would require additional follow-up with participants. Future work should also examine how this type of psychoeducation session relates to help-seeking behaviors specifically among African American women experiencing perinatal mood and anxiety disorders, as well as the reciprocal effects between changes in stigma of African American mothers and changes in stigma of their family members and other members of their support network.

The SATMIS (Kobau et al., 2010), while useful for measuring stigmatized attitudes, is limited by the lack of extensive validation with samples of African American women in the perinatal period and their support networks. Future work should include psychometric testing, including investigating measurement equivalence and/or differential item functioning in samples of African American women in the perinatal period and their support networks. Although comparisons to other racial/ethnic groups was not the focus of this study, understanding whether stigma can be measured similarly for African American mothers and other groups is necessary to improve health equity.

Future research should also refine the psychoeducational group used in this engagement project for large-scale testing with rigorous study designs such as randomized controlled trials with appropriate control/comparison groups. Other community health education groups could be used as comparison groups to control for non-specific intervention effects. Future trials should expand to larger samples of African American women, and eventually more diverse samples to increase external validity. Exploring differences between in-person and virtual/online delivery of the groups also seems to be a potentially useful contribution. Researchers should also incorporate longer follow-up periods along with assessments of help-seeking behaviors to determine whether reduced stigma translates to increased treatment use, as theorized. Doing so would provide stronger evidence for this psychoeducational group’s effectiveness in reducing mental health stigma and improving maternal health of African American women.

## Conclusion

Our engagement project demonstrates the value and preliminary effectiveness of a culturally tailored psychoeducation session for reducing mental health stigma among African American women and their social support networks. The large reduction in stigmatized attitudes observed after a brief, two-hour session confirms how targeted, culturally specific approaches to psychoeducation can meaningfully impact barriers to mental health care. African American women face disproportionate burdens of perinatal mood and anxiety disorders alongside persistent disparities of maternal mortality, making stigma reduction efforts particularly important. Educational approaches incorporating cultural context and community involvement represent a promising approach to improving help-seeking behaviors and maternal health outcomes in African American women.

## Data Availability

No datasets were generated or analysed during the current study.
